# Supplementary figures and images for: Comparative Transcriptomic and Functional Assessments of Linezolid-Responsive Small RNA Genes in Staphylococcus aureus
Source: mSystems. 2020 Jan 7;5(1):e00665-19. doi: 10.1128/mSystems.00665-19 (PMC6946794; doi:10.1128/mSystems.00665-19)

**Supplementary Figure 1**

**
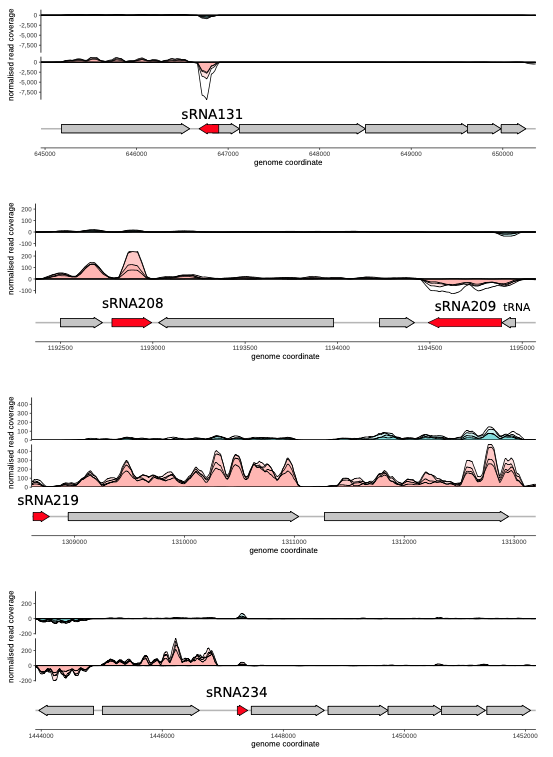
**

**
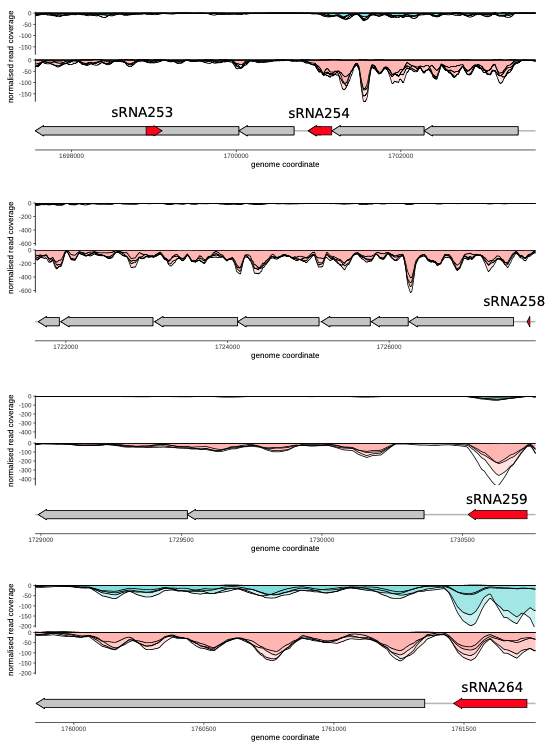
**

**
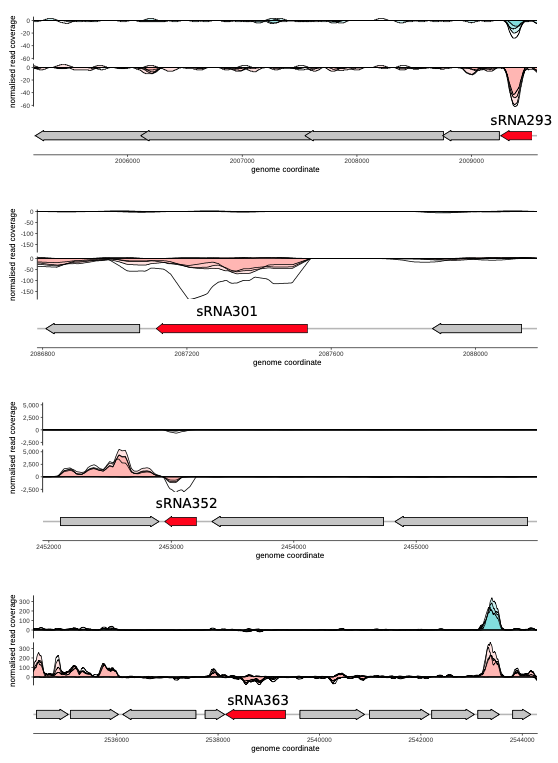
**

**
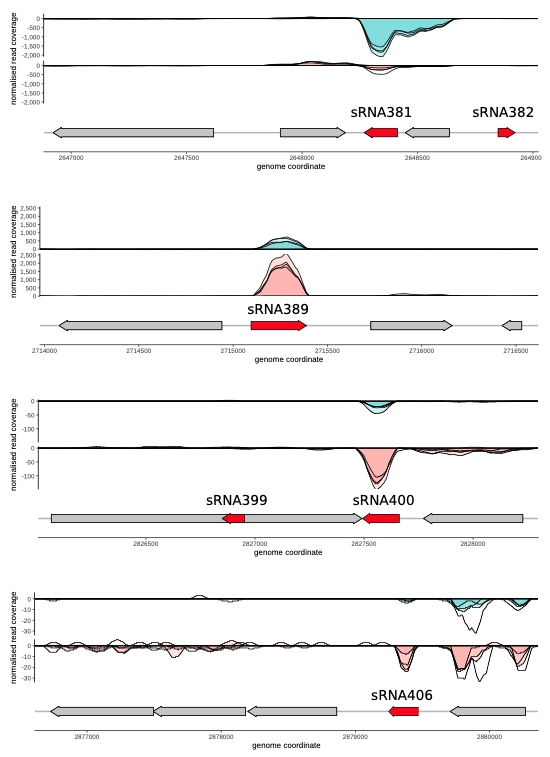
**

Supplement: FIG S1 [file mSystems.00665-19-sf001.docx]
